# Clinical, Safety, and Engineering Perspectives on Wearable Ultrasound Technology: A Review

**DOI:** 10.1109/TUFFC.2023.3342150

**Published:** 2024-07-09

**Authors:** Pengfei Song, Michael Andre, Parag Chitnis, Sheng Xu, Theodore Croy, Keith Wear, Siddhartha Sikdar

**Affiliations:** Department of Electrical and Computer Engineering and the Beckman Institute, University of Illinois Urbana–Champaign, Urbana, IL 61801 USA; Department of Radiology, University of California San Diego, La Jolla, CA 92093 USA; Department of Bioengineering, George Mason University, Fairfax, VA 22030 USA; Department of Nano and Chemical Engineering and the Department of Radiology University of California San Diego, La Jolla, CA 92093 USA; U.S. Army Medical Center of Excellence, Fort Sam Houston, TX 78234 USA. He is now an Independent Consultant; U.S. Food and Drug Administration, Silver Spring, MD 20993 USA; Department of Bioengineering, George Mason University, Fairfax, VA 22030 USA

**Keywords:** Quantitative biomarkers, ultrasonic imaging, ultrasonic transducers, wearable health monitoring, wearable sensors

## Abstract

Wearable ultrasound has the potential to become a disruptive technology enabling new applications not only in traditional clinical settings, but also in settings where ultrasound is not currently used. Understanding the basic engineering principles and limitations of wearable ultrasound is critical for clinicians, scientists, and engineers to advance potential applications and translate the technology from bench to bedside. Wearable ultrasound devices, especially monitoring devices, have the potential to apply acoustic energy to the body for far longer durations than conventional diagnostic ultrasound systems. Thus, bioeffects associated with prolonged acoustic exposure as well as skin health need to be carefully considered for wearable ultrasound devices. This article reviews emerging clinical applications, safety considerations, and future engineering and clinical research directions for wearable ultrasound technology.

## Graphical Abstract

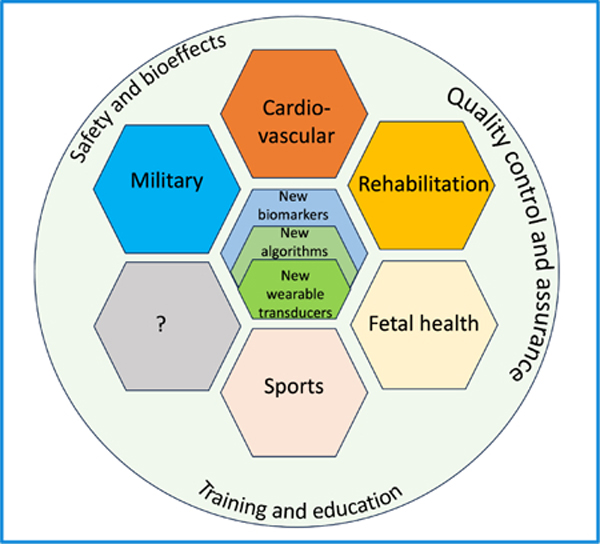

## I. Introduction

Over the past several decades, advances in microelectronics and material science have enabled continual miniaturization and enhancement of ultrasound (US) imaging systems, leading to profound changes in the application of diagnostic US in a variety of healthcare settings. In particular, low-cost and portable US imaging systems have enabled lifesaving diagnostic capabilities in low-resource settings [1]. Point of care US (POCUS) systems [2] have seen increased usage at the bedside [3] and in nonclinical environments such as a battlefield [4], natural disasters [5], public health emergencies [6], and space [7].

At present, the field of US imaging is poised for yet another potentially revolutionary advancement: wearable ultrasound [8], [9]. In contrast to POCUS, where the miniaturization happens at the scanner side (e.g., the main body of the US system), wearable ultrasound miniaturizes handheld transducers into wearable form factors, enabling real-time imaging in freely moving humans. Whereas traditional US has been used for diagnosis and image-guided interventions in radiology, cardiology, surgery, obstetrics and gynecology, emergency medicine and musculoskeletal imaging, wearable US has the potential for both clinical and nonclinical applications, such as cardiovascular, cerebrovascular, and fetal monitoring, rehabilitation, sports medicine and athletic training, military medicine and assistive technology. While the field of wearable US is still in its infancy, rapid technological advances are being made with exciting applications rapidly emerging. It is likely that commercial wearable US devices may soon become available to both clinical and nonclinical users. This new and rapidly evolving trend raises some important technical as well as open research questions surrounding the clinical usefulness, biosafety, quality control (QC), and other engineering considerations associated with wearable US. As such, the purpose of this perspective paper is to articulate some guiding principles for the future technical and clinical research directions involving wearable US.

This article is organized as follows. First, we outline several unmet clinical needs that could be addressed by wearable US technologies, including a discussion of potential challenges for adoption in different healthcare settings. Second, we discuss potential safety, bioeffects, and quality assurance considerations that are essential for the safe and effective use of wearable US, particularly under nontraditional and nonclinical settings by novice users of US. Finally, we share our perspectives regarding engineering research challenges that need to be considered for wearable US from the points of view of both hardware (e.g., probe design and fabrication) and software (e.g., beamforming, data analysis, and interpretation).

## II. Unmet Clinical Needs That Can Be Addressed by Wearable US Technology

The widespread clinical adoption of US has been driven in part by its relatively low cost and the advantageous design of US systems, i.e., a handheld transducer enabling interactive real-time imaging in a variety of settings. However, this design also presents inherent challenges [10]. These include operator dependence in selecting the anatomical imaging planes, the expertise required to interpret 2-D US images in relation to underlying 3-D anatomy, limited acoustic windows into the body, and a field-of-view limited to the position and orientation of the transducer. In addition, US has been challenged by difficulties in standardizing imaging during dynamic tasks and obtaining repeatable, reproducible, and reliable quantifications of physiological and tissue properties. Wearable US devices have the exciting potential to address many of these challenges, by eliminating handheld operation, enabling placement of multiple sensors simultaneously on different body areas, and achieving a long-sought goal of imaging dynamic function in freely moving humans. In the following subsections, we describe some of the current unmet clinical needs that can be addressed with wearable US imaging devices. Table I summarizes the example applications.

### A. Wearable US for Cardiovascular Applications

The American College of Cardiology, Washington, DC, USA, noted that one-third of global deaths in 2019 were attributed to cardiovascular disease and that the incidence of cardiovascular disease is rising [11]. US has important applications for establishing the health of a patient’s cardiovascular system as well as for the management of ill and postsurgical patients [12]. Despite many promising advances [8], clinical quality cardiovascular US imaging remains a challenge for wearable US devices. Transthoracic comprehensive echocardiography requires views of the complete heart that may not be feasible with a small wearable patch [12]. However, a promising goal for wearable US devices is to provide extended temporal monitoring of patients in and out of the hospital setting, for which it may be sufficient to focus on specific regions of interest or aspects of the heart function relevant to the clinical problem [13]. Wearable US devices may help reduce some sources of variability known to occur in echocardiography measurements [14]. For example, the assessment of recommended metrics such as left ventricular ejection fraction, diastolic volume, mass, and wall motion score with handheld probes may be subject to intra- and inter-operator variation largely due to positioning [15].

Imaging techniques in diagnostic US are based on mapping backscattered energy from tissue and interfaces, as well as mapping blood flow. Wearable devices have been successfully applied to common US imaging techniques. In A-mode imaging, 1-D data of echo amplitudes along a single line are recorded with depth. Motion of the interface echoes has been displayed by translating the 1-D A-line over time to record the relative motion of cardiac chamber walls or valve leaflets, known as M-mode. In B-mode US, 2-D maps of multiple lines of backscatter data are recorded as a cross-sectional plane with echo amplitude converted to a pixel brightness. Blood flow assessment can be performed in either continuous wave (utilizing the Doppler frequency shift principle with separate transmit and receive transducers) or pulse wave (utilizing motion-induced phase shifts between consecutive pulsed echoes) modes. In the latter case, color flow coding may be applied to the motion map superimposed onto the B-mode image, with red indicating motion toward and blue motion away from the transducer. Color power imaging sacrifices directional indication for improved sensitivity to slow flow.

**Fig. 1.**
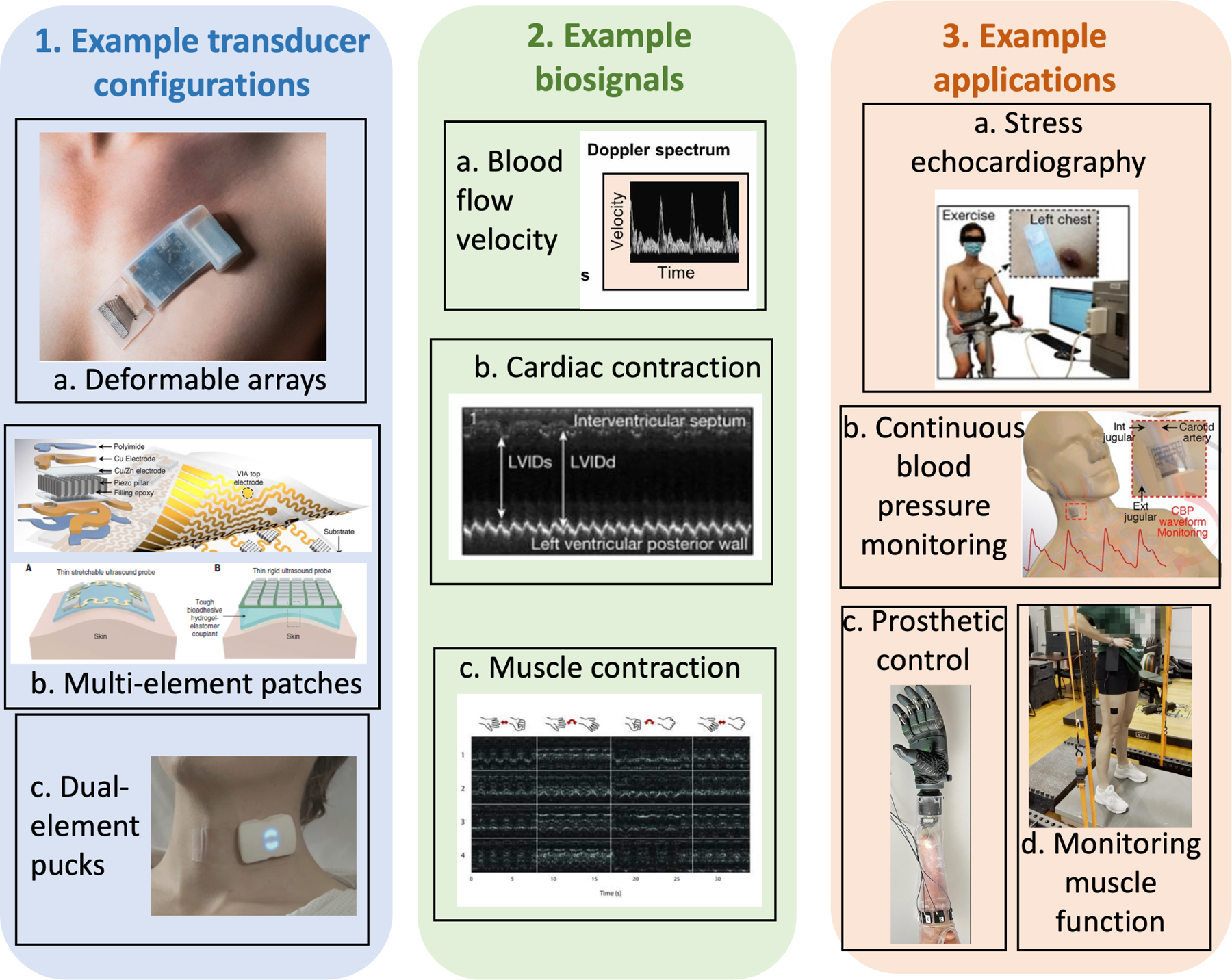
Overview of current state of the art for wearable US. Wearable US systems involve different configurations for transducers, such as deformable arrays [69], multielement patches [8], [18] and single-element pucks [70]; biosignals, such as Doppler spectra [8], cardiac contraction [19] and muscle contraction [53]; as well as applications such as stress echocardiography [8], continuous blood pressure monitoring [20], prosthetic control [60] and muscle function monitoring [70]. Research and development of integrated systems for specific use cases will require interdisciplinary expertise from a range of clinical, scientific and engineering disciplines. All figures were reprinted from their respective journals with permissions. 1. Example transducer configurations—(a) Deformable arrays. (b) Multielement patches. (c) Dual-element pucks. 2. Example biosignals—(a) Blood flow velocity. (b) Cardiac contraction. (c) Muscle contraction. 3. Example applications—(a) Stress echocardiography. (b) Continuous blood pressure monitoring. (c) Prosthetic control. (d) Monitoring muscle function.

Some of the most prominent applications for single-element wearable US are measurements of blood pressure waveform, heart rate, vessel diameter, and pulse wave velocity (arterial wall displacement). Continuous monitoring of blood pressure waveforms, derived from arterial wall displacement measurements, provides biomarkers to detect abnormal cardiac activities in the diagnosis and postoperative assessment of individuals at a high cardiovascular risk, Fig. 1, [16], [17], [18]. M-mode US is suitable for monitoring the structural dynamics of the heart walls and chambers, and cardiac output capacity over extended periods of time. An example of a wearable M-mode application for continuous monitoring of ventricular dimensions over time appears in Fig. 1 [8].

A wearable US patch attached to the neck was shown to be capable of continuously tracking flow parameters in the carotid artery [13], including continuous qualitative and quantitative tracking of pulsatile flow. A lightweight and wearable US sensor for inspection of blood flow velocities of deep arteries in real time has been developed [19]. In clinical practice, the patient’s blood flow velocities and assessments of these conditions are often required. Peak systolic velocity (PSV), end-diastolic velocity (EDV), time-averaged peak velocity (TAPV), and several flow velocity indices or ratios can be extracted and interpreted. The resistive index (RI) and pulsatility index (PI) are common indicators of vascular resistance and compliance. Pulsed-wave spectral analysis provides quantitative information about vasculature and vital data for identifying conditions that affect cardiovascular health. Color flow and color power imaging of the carotid artery have been demonstrated, comparing favorably to full-size clinical systems [13], [18], [19]. Wearable sensors are currently focused on monitoring blood flow using pulse-wave rather than continuous wave mode [8]. Flow velocity measurement and multimode Doppler are research priorities.

B-mode wearable US using array transducers has been used for imaging of the heart and vessels for conditions such as heart failure, pulmonary hypertension, cardiomyopathy, etc. Wearable US patches performed stably with body movements for continuous monitoring and compared favorably to clinical scanners [20]. However, echocardiography can be challenging in many patients with large body habitus, and contrast agents are currently used to improve image quality [21]. This may be a significant challenge for monitoring applications.

### B. Wearable US for Transcranial Doppler Assessment

Transcranial Doppler ultrasonography (TCD) is a non-invasive, bedside, portable tool for assessment of cerebral hemodynamics, detection of focal stenosis and arterial occlusion, monitoring treatment effects, and assessment of vasomotor reactivity [22], [23], as well as brain motion [24], [25]. Although uncomfortable for longer-term use, modern TCD head frames allow continuous hands-free emboli detection, providing risk stratification and assessment of treatment efficacy in several cardiovascular disease processes. TCD is an excellent screening tool for vasospasm in aneurysmal subarachnoid hemorrhage because of its high sensitivity and negative predictive value. TCD is used intraoperatively during carotid endarterectomy and stenting to provide hemodynamic management while minimizing the risk for brain ischemia [26].

Accurate and continuous monitoring of cerebral blood flow is valuable for clinical neurocritical care and fundamental neurovascular research. Continuous monitoring of cerebral hemodynamics enables screening for and diagnosis of brain disorders (e.g., vasospasm, stenoses, aneurysms, and embolisms) as well as understanding neurological neurovascular functions (e.g., sensory, motor, and cognitive controls) [27]. However, to examine relevant vasculature requires two-way transit across the skull and brain, presenting challenges to Doppler analysis due to signal attenuation and aberration [28]. Conventional TCD probes are rigid and need to be either handheld or tightly fastened with a headset. Operator-dependency, subjective selection of the target arterial segment and user discomfort limit the measurement accuracy and practicality for prolonged recording [29].

The development of wearable devices for TCD addresses many of these constraints through electronically steered velocimetry systems, which enable noninvasive measurement of cerebral blood flow velocity (CBFV) with limited operator interaction. Wearable prototype systems for continuous measurement of CBFV that are capable of autonomous vessel search and tracking have been reported [29]. Although flow phantom and preliminary human validation show promise, further testing is needed for comparison to existing commercial TCD systems. Validation is needed for vessel identification and tracking in a variety of patients and under head movement and ambulation. Detection of transient emboli in the blood with Doppler spectrum analysis has been proposed even for surgical environments [30], [31].

Another unexplored opportunity for wearable US may be in monitoring brain acceleration and the impact of shock wave propagation through the brain [25] during contact sports, as well as in the battlefield, to better understand the impact of concussion and mild traumatic brain injuries. Despite the increasing use of sensors and other approaches to track impact of the head in athletes [32], there is currently no feasible method for directly measuring brain motion. This has been a long-standing challenge in the field of concussion biomechanics. Wearable US may provide an opportunity to achieve this goal since transcranial US provides an opportunity to directly measure brain motion. However, many technical challenges need to be overcome, due to acoustic attenuation and phase aberration introduced by the skull, before the feasibility of this approach using wearable devices is demonstrated.

### C. Wearable US for Fetal Monitoring

Despite advances in medicine, childbirth mortality is a major issue worldwide [33]. A key factor for the prevention is early detection of fetal compromise. Fetal movements in utero are early expressions of fetal neural activity and are indications of fetal well-being. A healthy fetus moves regularly until labor, so changes or disappearance of fetal movements are indications of potential fetal compromise [34]. It is also observed that fetal movements are reduced in cases of placental insufficiency [35]. In high-risk pregnancies, especially when placental insufficiency is suspected, monitoring of fetal movements would be valuable.

Existing clinical methods of monitoring fetal movement include maternal perception and sonographic evaluation of movements of fetal trunk, limb, or head [36]. Fetal sonography is the preferred clinical tool; however, it is generally performed only by expert operators in hospital or outpatient clinical settings, thus it may not be available to all patients at the needed times during pregnancies. Maternal perception of fetal movements as early as week 25 of pregnancy is widely employed and does not require a clinical setting [33]. The major drawback of this method is its subjectivity; it is reported that the sensitivity of maternal perception can vary considerably [36], [37].

Fetal heart rate (fHR) monitoring using Doppler US is a reliable method to assess fetal health before and during labor. However, it only allows short or intermittent assessment and is operator-dependent [35]. During labor, continuous monitoring of fHR is valuable especially for high-risk deliveries. Continuous monitoring of the fHR is performed using a US transducer fixed on the maternal abdomen and may be combined with simultaneous monitoring of the uterine activity, known as cardiotocography. The procedure has known limitations including periods of signal loss and inaccurate estimation of fHR. Signal loss may occur in premature deliveries, mothers with high BMI, and multiple gestations [38], or simply positional shifts of the transducer or fetus.

Wearable US systems have been demonstrated to accurately record Doppler waveforms at frequencies appropriate for in utero use [36]. Applications in nonclinical settings would make them accessible and affordable in underserved communities where most stillbirths occur. fHR monitoring and labor surveillance are identified as keys to prevention [39].

A flexible US transducer array was proposed that allows for measuring the fHR independently of knowing the fetal heart location (fHL) [40]. In addition, a method for dynamic adaptation of the transmission power of this array was introduced with the aim of reducing the total acoustic dose transmitted to the fetus and the associated power consumption, an important design requirement. The transducer array correctly measured the fHR for varying fHL while only using 50% of the total transmission power of standard clinical US transducers.

### D. Wearable US for Musculoskeletal and Rehabilitation Applications

Musculoskeletal rehabilitation applications of wearable US may usher in a new era of in vivo imaging during exercise [41], [42], rehabilitation [43], or even sport performance tasks after injury. Current technical challenges of clinical musculoskeletal US imaging include maintaining probe position, adequate field-of-view, and sufficient skin contact during dynamic (but slow) subject movement during an imaging examination. A wearable US with miniaturized and adhesive sensors or arrays may improve muscle activity monitoring, tendon loading, or ligament stress under controlled clinical conditions designed to test muscular performance or tendon loading [44].

Rehabilitation clinicians are interested in ensuring post-injury muscle recovery including the ability to contract, control, and engage synergistic muscle activity to achieve optimal, pain-free movement consistent with the planned rehabilitation goals. Clinical information consisting of patient self-report, visual exercise performance, and palpation skills [45] may be augmented further with wearable US to provide biofeedback of quantifiable muscle activity during an entire rehabilitation session, enabling quantification of load, progress over time, or even insufficient effort [44]. Identification and prevention of arthrogenic muscle inhibition following common knee injuries have been found to be an important rehabilitation priority in athletes making advancements in muscle activity monitoring consistent with current clinical recommendations [46], [47]. Wearable US may play an important role in informing clinicians and patients about muscle function over time.

Unlike the other systems, the musculoskeletal system’s primary function is the production and execution of movement and stability of the body, making clinical understanding of tissues during movement a key concept to understand [48]. While few imaging applications enable imaging during movement, wearable US could unlock further knowledge of tissue behaviors under increasingly dynamic conditions and spark new approaches to rehabilitative treatment of muscle, joint, or tendon injuries [44]. Unquestionably, dynamic imaging is challenging, leaving clinicians with reasoned assumptions on musculoskeletal function during dynamic tasks. Advances in video-based evaluation and coaching methods have improved 2-D and 3-D assessment of rapid, multiplanar movement. Wearable US may augment clinical observation and coaching to further enable rehabilitation progress and improvement by providing real-time biofeedback associated with the activation of specific musculoskeletal tissues during rehabilitation exercises.

Surface electromyography (sEMG) has been used for sensing electrical activity of muscles during rehabilitation [49], in addition to joint kinematic measures. The ability of a muscle to generate force depends not only on electrical activation but also on changes in muscle tissue properties, such as fibrous and fatty infiltration [50], and the biomechanical properties of the connective tissue (fascia) that are critical for force transmission from muscle to joints [51]. Wearable US can image muscle architecture and tissue properties, as well as muscle mechanics [44]. Thus, wearable US can provide distinct and complementary information compared to sEMG and other conventional joint-based biomechanical measures.

Periodic application of wearable US technology could be used during rehabilitation sessions in the early (0 to 6 weeks postoperative or postinjury) to help monitor and re-establish quadriceps activity prior to applying more advanced strengthening exercises. Repeated isometric or isotonic muscle contractions using wearable US can be used to quantify patient effort, consistency, and limb symmetry in order to inform patient and clinician about the effectiveness of the exercise early in postoperative or acute recovery phases with reasonable accuracy [41]. New opportunities for developing biomarkers to inform exercise dosing may emerge, which could include contraction strength, intensity, or hold duration. Visual observation of exercise performance could be correlated with wearable US feedback of muscle activity to understand fatigability limits or tendon loading [52] in order to alter the session according to the clinician’s interpretation. These metrics derived from wearable US devices could result in targeted goals quantifiable at the level of individual muscles, which is expected to be a significant improvement over patient self-report alone.

Rehabilitation following nerve injury, limb amputation, or stroke may be enhanced via specific biofeedback of muscle groups or proximal muscles during the relearning of motor patterns to facilitate early rehabilitation. Real-time and continuous biofeedback enabled by wearable US monitoring could enhance patient performance and provide tangible outcomes facilitating further clinical goal setting. Visual or auditory feedback of muscle activity can facilitate clinical teaching and enable a patient to initiate or correct a movement pattern consistent with the clinician’s directions. Neurological rehabilitation involving patients with hemiparesis may be augmented through continuous monitoring of select muscles during functional activities (such as ambulation). Wearable US-based outcome measures may include muscle activation patterns, bilateral asymmetry of activation, and changes in activation patterns over time during a course of care.

Baseball pitchers and batters, golfers, or athletes skilled in repeatable movements requiring consistent starting and ending positions may benefit from monitoring of core musculature via wearable US sensors to identify core muscle activity necessary to initiate consistent, rapid trunk movements. Core stabilization is known to be necessary prior to movement initiation, however, lumbar spine injuries have been shown to impair core muscle activation and timing [53]. Muscular activation patterns of specific and repeatable movement tasks (gait, pitch, golf swing) could be integrated into video recording of those tasks to develop a deeper understanding of movement pattern characteristics observed by therapists, coaches, or trainers and may lead to novel approaches to movement correction and rehabilitation provided that the device allows free movement.

### E. Wearable US for Rehabilitation Engineering and Neuroengineering Applications

A novel application of wearable US that is being investigated involves sensing of muscle activity to infer the volitional intent of a user with neuromuscular and motor impairments, such as amputation [54], stroke, and spinal cord injury [55]. Reliable decoding of the user’s intent can enable the volitional control of an assistive device such as a powered prosthetic hand or a leg, or an exoskeleton. However, decoding the user’s intent has been a major challenge in the fields of rehabilitation engineering and neuroengineering [56]. For the past 50 years, the dominant method of decoding volitional intent from muscle activity has involved the use of sEMG sensors that detect the electrical activity associated with muscle contraction. However, sEMG has many limitations [57]. These include the inability to resolve the source of activation when multiple muscles overlie each other and to distinguish relative muscle firings between adjacent muscles. In addition, sEMG is unable to reliably monitor deeper muscles and has somewhat poor signal-to-noise ratio (SNR), especially for weak muscle contractions, and suffers signal degradation due to muscle fatigue. Therefore, many of the recent advances in neurorobotics have involved implanted sensors, such as implanted myoelectric sensors, to improve spatial specificity and SNR. Recent advances in wearable US have opened up a new method of inferring volitional intent by analyzing the mechanical deformation of muscle during contraction. This method is known as sonomyography (SMG). SMG has advantages over sEMG, including spatial selectivity enabled by the depth resolution of US, as well as potentially superior SNR [58]. US can also be used to analyze muscle twitch response during functional electrical stimulation, which is not practical with sEMG [59]. New research has also shown the ability of US to isolate spatially-resolved motor unit action potentials [60]. SMG has been demonstrated to be feasible for controlling both upper [61], [62], [63], [64] and lower [65] prosthetic limbs. In addition, preliminary work is demonstrating the ability for SMG to decode the intent of users with spinal cord injury [66]. Rapid advances are being made in this novel application of US, and wearable US technology can hasten the translation of these advances to benefit patients with motor impairments. There are also opportunities to combine SMG and sEMG to impact of electromechanical delay (EMD) [67], and better predict muscle force development and joint kinematics [68].

### F. Potential Barriers to Clinical Adoption

Smart wearables generate a plethora of data through various sensors and software algorithms. Understanding their basic engineering principles and limitations can be helpful for clinicians and scientists. Several challenges still hinder the widespread adoption of wearables in clinical practice, including a concern for device accuracy, data security, patient privacy, cost, and how to separate actionable data from noise.

Wearable B-mode US sensors have been used to measure many tissues in the human body, but there are still a number of issues that need to be addressed [9], [72]. For example, measurement accuracy is constrained by the robustness of continuous acoustic coupling between the sensor and the skin, especially during free movement. In addition, the acoustic field of a flexible array may change when it is worn in different locations on the body, which poses a challenge for the controllability of the acoustic field. The varying curvature of the skin also affects detection capability of a wearable US array, although different beamforming strategies may correct for this.

Evidence supports the use of wearable devices in cardiovascular risk assessment and cardiovascular disease prevention, diagnosis, and management [73], but large, well-designed clinical trials are needed to investigate their potential advantages over existing standard of care.

Potential reluctance to learn and experiment with wearable US devices may impair the widespread clinical adoption of wearable US. Perceptions of setup time and device complexity by clinicians or patients may delay the adoption, as would anything requiring interpretation of US images, issues already known to be barriers in the adoption of POCUS [74]. Long-term use of wearable US will raise concerns for skin damage, particularly in the elderly population, and early studies must investigate the correlation between wear time and skin health [75]. Device purchase and maintenance costs should be considered from the end-user perspective including administrators involved in purchasing decisions who must weigh the perceived benefit of the output against the complexity of integrating wearable US into patient care, including changes to workflow, personnel training, etc.

There are many opportunities to enhance the performance of wearable acoustic sensors in terms of sensitivity, specificity, response time, and power consumption. Advances include the use of manufacturing techniques, such as piezoelectric micromachined ultrasound transducers (PMUTs) [76], as well as novel piezoelectric materials [77]. The sensor size, shape, as well as the choice of backing and matching layer can impact sensor performance [78]. Machine learning algorithms can be used to analyze sensor data, extract useful information, and potentially provide rapid feedback to adjust beam steering and power [79]. Noise reduction techniques can be used for better SNR and to improve the sensor’s sensitivity. In a stretchable and bendable sensing system, mechanical stresses due to the movement of the human body can cause displacement of the components, which poses a significant challenge to wearable electronics. Motion artifacts can introduce large noise that compromises the system measurement. Although challenging, wearable acoustic sensors could be integrated with other types of markers and systems, such as integrated laser scanning [80], and strain sensors [81] to allow updating the locations of individual transducer elements once applied to the patient.

Educating the users will be a significant prerequisite for successful adoption. Preparing a practical guide for clinicians can facilitate the integration of these devices in routine clinical practice. Clinicians should be involved in the development and dissemination of appropriate guides, through hands-on-workshops and other training activities through professional societies.

## III. Safety Considerations For Wearable US Devices

### A. Potential Bioeffects of US

Potentially harmful effects of US on tissue (i.e., bioeffects) are generally divided into thermal and nonthermal. Nonthermal bioeffects, such as cavitation, are generally thought to be threshold phenomena that occur when a particular level of acoustic output is exceeded [82], [83]. Thermal bioeffects tend to be cumulative in nature, depending on exposure duration as well as instantaneous acoustic exposure. Wearable US devices, especially monitoring devices, have the potential to apply acoustic energy to the body for far longer times than would typically be encountered in conventional diagnostic US exams. Therefore, this section will emphasize safety considerations associated with long exposure times.

**Fig. 2.**
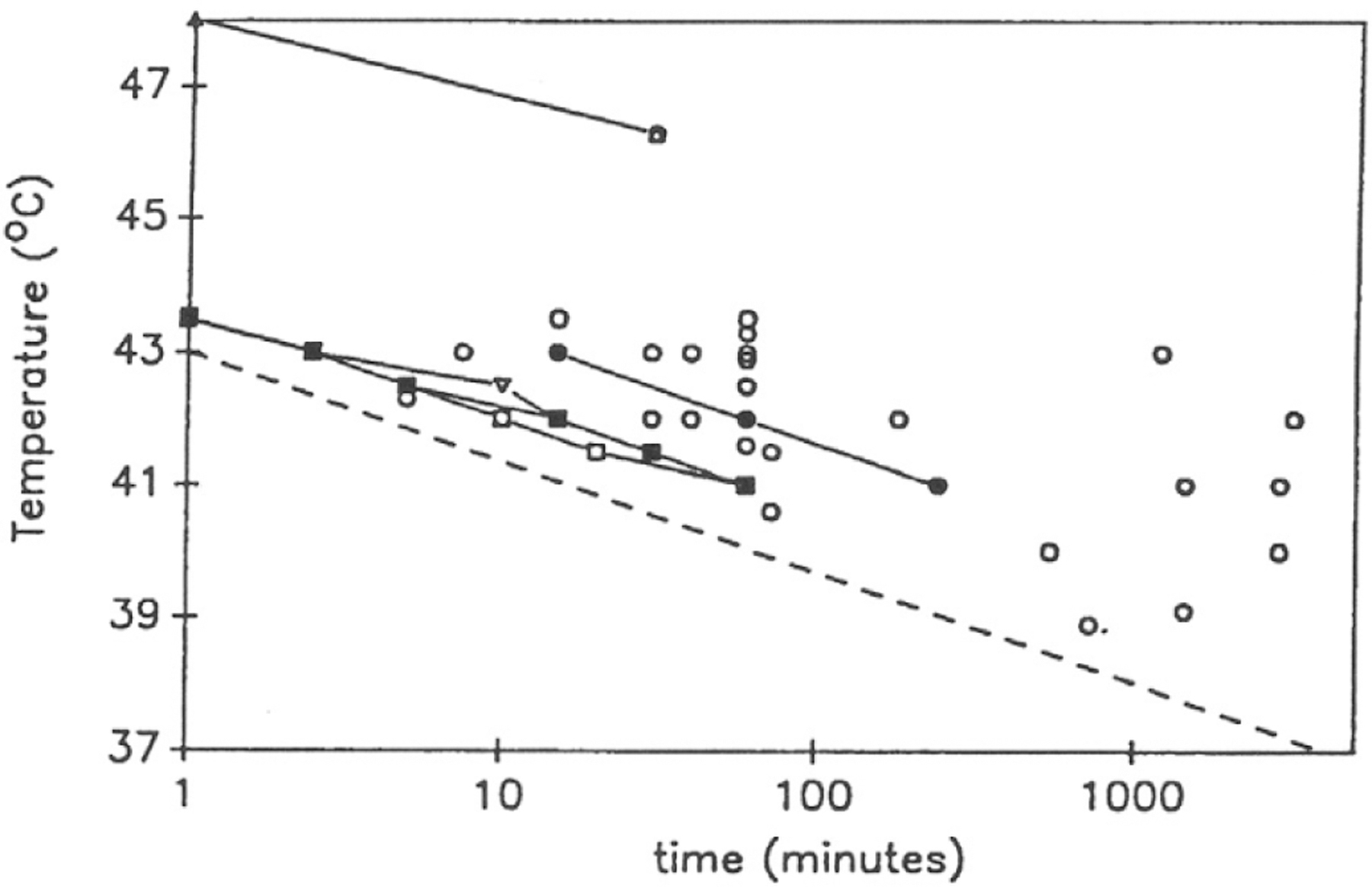
Thermal Bioeffects. A plot of thermally produced biological effects that have been reported in the literature in which the temperature elevation and exposure durations are provided. Each data point represents either the lowest temperature reported for any duration or the shortest duration for any temperature reported for a given effect. The solid lines represent multiple data points relating to a single effect. Combinations of temperature and exposure duration above the dashed line are considered unsafe. Reprinted from [85] with permission from Elsevier.

When US is applied to tissue, a fraction of the applied energy is absorbed by the tissue and converted into heat. Sufficiently large quantities of heat can potentially damage tissue. The likelihood of thermal bioeffects in tissue due to any heat-generating source depends on the magnitude and duration of elevated temperature. There are abundant empirical data from experiments with heat applied to mammals to indicate combinations of temperature rise and exposure duration that give rise to bioeffects [84], [85], [86], as shown in Fig. 2.

The actual temperature rise induced by US in humans in vivo is usually unknown. Temperature rise could be measured by inserting a measurement device such as a thermocouple into the tissue, but this would generally be unacceptably invasive. Temperature rise could be measured using magnetic resonance thermometry, but this would usually be impractical as it would require the subject to lie in a magnetic resonance imaging system during US exposure, completely defeating the purpose of a compact, portable, wearable US device.

### B. Safety Considerations for Wearable US

These practical limitations can be avoided by using an estimate of temperature rise called the thermal index (TI). The TI was developed to provide a real-time, onscreen index of the likelihood of thermal bioeffects to guide diagnostic US scanner operators during clinical exams. The TI for a given transducer under specified driving conditions (e.g., transmit voltage waveforms and beamforming algorithms) may be computed from acoustic output parameters (i.e., power and intensity) that may be measured in a water tank during system calibration. The effects of tissue attenuation in vivo may be accounted for by applying an exponentially decaying “derating” function to the measurements. Under a set of assumptions, including values for tissue attenuation and absorption properties, the TI corresponds to the temperature rise in degrees Celsius induced in the tissue by the US pressure waveform [87]. However, since tissue attenuation and absorption properties can vary substantially from tissue to tissue and from subject to subject, the TI is usually regarded as just an approximate estimate of temperature rise.

Since bone has a much higher absorption coefficient than soft tissues [88], the presence of bone in the US propagation path can have a big influence on expected temperature rise. Therefore, there are three models for TI to accommodate various situations that might be encountered in vivo: soft tissue (TIS), bone at focus (TIB), and bone (i.e., cranium) at surface (TIC) [87]. Depending on the transmitted pressure waveform, maximum tissue heating can occur near the skin surface or deeper into the tissue. In addition to dependence on acoustic output, TI can depend on aperture size and frequency. More detail on measurement methodology for TI is provided in an International Electrotechnical Commission (IEC), Geneva, Switzerland, standard [89] (IEC 62359, 2017). Beam intensity can be considerably underestimated due to hydrophone “spatial averaging” that occurs during the calibration process. Spatial averaging can lead to underestimation of TI, particularly for highly focused, nonlinear beams such as pulsed Doppler and acoustic radiation force impulse (ARFI) waveforms [90], [91].

Based on empirical data for thermal bioeffects in mammals, professional organizations such as the American Institute of Ultrasound in Medicine (AIUM), Laurel, MD, USA, and the British Medical Ultrasound Society (BMUS), London, U.K., have developed guidelines for maximum exposure durations as functions of TI [92], [93], [94], [95]. The guidelines are different for fetal and postnatal tissues because fetal tissues are more thermally sensitive than postnatal tissues. AIUM recommendations are shown in Table II.

Skin heating is a concern for all medical devices that contact the skin. Skin heating might be of particular importance for wearable US devices, considering the relatively long durations that they could be used. An IEC standard presents tests for estimating skin heating by medical US devices [96] (IEC 60601-2-37, 2015). One test involves applying the US device to a test object that mimics acoustic attenuation, specific heat capacity, and thermal conductivity of the tissue(s) that the device is intended to contact. Another test involves operating the US device against still air. Temperature of the transducer assembly may be measured during these tests, for example, with infrared radiometry or thermocouple methods. According to the IEC standard, the tests should be conducted for durations up to 30 min. However, the tests might not have envisioned devices with operating durations that might be intended for some wearable US devices. The standard provides acceptable temperature rises associated with these tests. IEC is currently working on a new standard to replace the section of IEC 60601-2-37 that applies to temperature rise measurements.

Skin irritation is a potential concern for all medical devices that contact the skin. Again, skin irritation might be of particular importance for wearable devices, considering the relatively long durations that wearable devices could be used. Many devices are encapsulated with silicone, which is considered a biocompatible material [97]. Polylactic acid (PLA) and poly(lactic-co-glycolic acid) (PLGA) are biodegradable and bioresorbable polymers extensively employed in medical applications, such as sutures, implants, tissue engineering scaffolds, and pharmaceuticals. However, their utilization in wearable electronics is limited, primarily due to the need for polymers with prolonged durability in sweat and biofluids. In the realm of wearables, silicone elastomers, polyurethane, and styrene-ethylene/butylene-styrene are prevalent choices. These polymers are known for their biocompatibility, making them suitable for on-skin applications, a fact well-documented in the literature [98], [99], [100].

More information on standards for biocompatibility may be found in ISO 10993-1 [101], and standards to ensure thermal, mechanical, and electrical safety of medical electrical equipment may be found in ANSI, Washington, IEC 60601-1-2 [102].

## IV. Engineering Considerations for Wearable US Devices

### A. Probe and Device Design

While the fundamental building blocks of soft, wearable US probes are reminiscent of conventional handheld probes [103], [104], their engineering necessitates certain compromises that impact their performance compared to the latter. Specifically, to achieve flexibility and stretchability, the transducer element pitch must be increased, resulting in degraded focusing capability and lateral resolution, and perhaps increased effects of grating lobes. Moreover, the packaging materials used for flexible/stretchable probes, typically based on silicon-elastomers, exhibit higher acoustic attenuation compared to those employed in conventional handheld probes. Consequently, this leads to weaker transmitted signals and lower SNR in the acquired signals. Additionally, the lifespan of flexible/stretchable probes is significantly shorter than that of conventional handheld probes, which can last for years when used and maintained appropriately. The former tend to experience wear and tear after repeated use due to their susceptibility to mechanical deformations.

Therefore, there exists a tradeoff between the performance of the probe and its flexibility/stretchability. The stretchability of human skin varies based on several factors, such as age, gender, ethnicity, and body location, but typically does not exceed 30% [20], [105]. For most applications, a wearable US probe with a stretchability in the range of 10%–30% would be sufficient. For scenarios where the probes should be flexible but need not be stretchable, another approach of bonding diced piezoceramic elements on a thinned complementary metal-oxide semiconductor (CMOS) device provides an innovative solution for integrating electronics with the transducer array [106], [107], [108]. This approach has been shown to produce strong US signal amplitudes amenable to diagnostic and therapeutic applications [106], [107], [108].

Although the goal is to maximize the performance of the wearable US probe, it is likely to remain challenging to match the capabilities of conventional handheld probes in the foreseeable future. However, it is important to note that wearable and conventional probes cater to different use scenarios. Wearable probes are well-suited for situations outside the hospital setting or in resource-limited areas where access to high-quality ultrasonography is limited. While they may not match the performance of conventional handheld probes, wearable probes can offer sufficient capabilities to monitor, screen, or triage subjects before they are referred to hospitals for further diagnosis [6], [8], [69], [109], [110].

Most of the current wearable US devices are wired for power supply and data transmission. However, there has been a recent demonstration of a fully integrated wireless wearable US device [69]. It is important to note that this wireless device is limited to A-mode, M-mode, and B-mode, which are less power-hungry and require less data compared to Doppler-mode imaging.

To progress toward a wireless Doppler-mode wearable US device, each acoustic channel will produce data at a substantial rate of tens of hundreds of megabits per second. To manage power consumption and data demands, we can implement multiplexing and duty-cycling during the transmit and receiving sequence. By doing so, power usage and data transmission can be reduced at the cost of temporal resolution. Given the array’s numerous channels, the power budget is estimated to range from tens to hundreds of milliwatts. To achieve real-time imaging capabilities, high-speed gigabit wireless network connections like 802.11ac may need to be employed [111]. For added convenience and versatility, the device can store the recorded raw radio frequency data onboard in a memory, allowing for later retrieval and further analysis on a terminal device.

### B. Equipment and Imaging Quality Assessment

Standards for QC of US imaging systems are provided by the ACR-AAPM technical standard (the American College of Radiology (ACR), Reston VA, USA; the American Association of Physicists in Medicine (AAPM), Alexandria, VA, USA) for diagnostic medical physics performance monitoring [112], the AIUM routine quality assurance guidelines [113], and IEC standards [114], [115], [116]. Clinical US equipment is subject to the established standards of acceptance testing, performance evaluation, and continuous quality assurance programs with periodic QC measurements. General purpose tissue-mimicking phantoms with acoustic targets of various sizes, contrasts, echogenicity, and locations are commonly used in QC for B-mode imaging, which includes spatial resolution (axial, lateral, and elevational), contrast resolution, geometric accuracy, uniformity, and depth of penetration [117]. For systems with Doppler capabilities, specific measurements and phantoms are available for assessing Doppler sensitivity and verifying flow velocity and direction accuracy [112], [117]. For strain elastography (SE), Dietrich et al. [118] serve as a general reference for quality assessment. For shear wave elastography (SWE), the Quantitative Imaging Biomarker Alliance of the Radiological Society of North America, Oak Brook, IL, USA, has provided consensus standard approaches for equipment calibration and imaging performance assessment [119], [120].

In principle, wearable US systems should be subject to the same standards for equipment and imaging quality assessment as conventional US imaging systems. However, since wearable devices are likely to be used in unconventional imaging settings, QC for wearable US presents unique challenges. For example, routine system checks and cleaning that are recommended on a daily basis and regularly conducted by sonographers, physicians, or other qualified US system users may not be possible when the device is deployed in a non-clinical environment. Since daily routine checks and cleaning involve inspections for transducer damage (e.g., cracks, separations, and imaging artifacts associated with dead elements or a delaminated lens) and transducer disinfection, missing these tasks may compromise the fidelity of the system and impose health risks to patients, especially when the device is attached to body surface for sustained periods of time. While transducer disinfection and physical damage inspections (e.g., cracks, discolorations) may be carried out routinely by patients, imaging quality assessment is challenging for novice users of US systems who are also unlikely to have access to standard US QC phantoms. This issue is further compounded by the delicate wearable transducers which tend to be more susceptible to physical deteriorations and damage as compared with conventional handheld transducers. Meanwhile, since wearable US devices are likely to be used for continued monitoring of small tissue variations that sometimes occur during long periods of time, assurance of imaging reproducibility becomes essential. This makes frequent and routine QC extremely important for wearable US systems. In the future, automatic and intelligent tools for transducer inspection (e.g., by using photographs of the transducer) and system-defect-related imaging artifact detection may be necessary to serve as an alternative to routine QC by practitioners. Electronic methods, internal to the wearable US system (e.g., by impedance spectroscopy), may be preferable to conventional phantom-based methods for identifying dead elements in the transducer array. Guidelines will also need to be updated to provide standards for QC of wearable devices used in unconventional imaging settings.

### C. Imaging Modes and Beamforming

To date, the majority of the reported wearable US devices support A-mode, M-mode, and B-mode imaging [8], [18], [69], [81], [105], [110], [121], [122]. Blood flow imaging (e.g., color flow and color power imaging), tissue Doppler, and SE have also been recently demonstrated [105], [110]. ARFI-based SWE (ARFI-SWE) has not been demonstrated on wearable devices yet due to the requirement of high-power “push” beams. In general, the imaging quality of existing wearable US devices is inferior to that of clinical US systems operating with traditional, handheld transducers. As discussed above, this limitation is largely attributed to the stretchable transducers, which suffer from weak transmissions, noisy reception, and other design compromises such as larger element pitch, leading to suboptimal imaging spatial resolution, contrast resolution, grating lobes, and SNR. The suboptimal signal quality from wearable transducers also negatively impacts US sensitivity to blood flow and tissue motion, which undermines Doppler and elastography performance [123]. Considering the fact that most existing wearable transducers are wired to a US system that is not wearable (e.g., Verasonics, Kirkland, WA, USA), true wearable devices (i.e., wireless and stand-alone) are expected to have further reduced imaging quality because of the power and data communication constraints [69]. As such, one should not expect wearable US devices to have comparable imaging quality as conventional clinical systems or the ability to replace them for established clinical applications. Instead, wearable US should be expected to provide sensitive and accurate detection of tissue variations compared to baseline and generate actionable notifications to users and care providers.

Similar to conventional US systems, delay-and-sum (DAS) beamforming is the most commonly used image reconstruction method for wearable US devices that involve B-mode image reconstruction using array transducers [8], [69], [110], [122]. In fact, most existing wearable array transducers are connected to Verasonics US systems and utilize Verasonics’ pixel-oriented software beamformer for image reconstruction [124]. FPGA-based hardware beamformers have also been reported for wearable US devices [81]. Some studies have explored nonlinear beamformers such as delay-multiply-and-sum (DMAS) to reduce incoherent imaging artifacts and improve image quality [69], [103]. For transmit sequences, synthetic aperture imaging, line-by-line scanning, and compounding plane wave imaging have all been reported [8], [69], [103]. Because of the relatively low SNR associated with wearable transducers, it is challenging to recover high-quality images via beamforming. However, advanced deep learning-based US beamformers and signal processing techniques [79], [125], [126], [127] may be used to mitigate low SNR using a data-driven approach. In addition, the movement of the wearable transducer elements responding to tissue deformation manifests as a phase aberration correction problem, which is mathematically analogous to uncertainty in ultrasound propagation delays due to aberrating layers (e.g., fat or skull), a longstanding issue of US imaging. Conventional, model-based phase aberration correction techniques typically require a “guide star” and computationally expensive algorithms to operate [128], [129], [130], [131], which is pragmatically challenging for real-time imaging applications. To address this challenge, transducer element positions may be actively measured (e.g., provided by embedded sensors in the wearable transducer) for beamforming or used as prior information for phase aberration correction. Advanced phase aberration correction techniques based on differential beamformers may also serve as promising solutions for fast and robust element position correction in real-time imaging with flexible devices [132], [133]. Meanwhile many deep learning-based phase aberration correction techniques have been rapidly emerging [131], which presents many enticing opportunities for addressing the unique problem of transducer deformation in wearable US devices.

### D. Data Analysis, Interpretation, and Education

A large number of transducer elements and associated electronic channels, megahertz sampling, and long-term monitoring applications can produce large volumes of data, especially for wearable devices designed for 3-D imaging. Sparse arrays and Fourier-domain convolutional beamforming [134] can be used for reducing both the channel count and sampling rate for US devices that rely on wireless data transmission and this approach was recently demonstrated to reduce the sampling rate by over a factor of 30 compared to standard DAS approaches [134]. Discrete cosine transforms have also been used for compressed sensing of US signals to facilitate miniaturized and portable US devices [135]. Deep learning is yet another emerging approach for obtaining high-quality US image reconstruction from subsampled US data [136], [137] without compromising imaging quality, albeit these methods often require large amounts of high-quality training data to produce generalizable models. Another promising approach that is particularly well-suited to reduce channel count in 2-D transducer arrays is row-column addressing in transducer arrays [138]. When implemented with orthogonal row-column electronic scanning and spatial compounding, these transducers have the potential to enable wearable devices for 3-D and ultrafast imaging [139].

Continued advances in reducing array channel counts, sub-sampling, or compressed sensing, and artificial intelligence are crucial for furthering the development of wearable US systems for widespread implementation. Wearable US imaging devices also have the potential to make this diagnostic technology more widely available to untrained or minimally trained users, provided that tools are developed for automated or easy image segmentation, labeling, and interpretation. Deep learning has emerged as a useful tool for addressing these needs in clinically relevant settings [140], [141], [142], [143], and these techniques are now being adopted in portable and wireless US systems [144], [145]. A key roadblock will be the availability of high-quality annotated US data for training deep learning networks. Efforts to make large quantities of US image data publicly available [146], [147] will facilitate training these deep learning networks.

## V. Summary: Guiding Principles for Future Research and Development

While we anticipate that future technical advances will continue to rapidly drive down the size and power consumption and increase the functionality of wearable US devices, we believe the following guiding principles could lead to successful translation and adoption of this technology in the near future.

1. In contrast to conventional diagnostic US systems where there is a high emphasis on image quality, wearable US systems need to generate robust and actionable quantitative measures for the end users. Thus, there is a need for an additional effort toward deriving and validating quantitative, reproducible, sensitive, and useful biomarkers from US data for quantifying tissue properties, physiology, and function.
2. Diagnostic US can be a safe imaging modality as long as ALARA (expose As Low As Reasonably Achievable) principles are followed. The applications for wearable US may involve exposures for longer durations than traditional diagnostic scans, and thus system designers need to be aware of potential bioeffects associated with prolonged US exposure and include mitigating measures (e.g., alternating between on and off modes) in the system design. ALARA guidelines need to be developed for wearable US applications and users need to be educated.
3. Current diagnostic US relies on the interpretation of images by trained experts. The potential applications for a wearable US will often involve users who are not trained sonographers. In these cases, quantitative image outputs of wearable devices should be tailored to be easily interpretable.
4. New QC and assurance guidelines need to be developed for wearable US systems that are likely to be distinct from the existing guidelines for diagnostic US systems and transducers. Manufacturers of wearable US systems need to develop simple and straightforward QC and quality assurance approaches that can automatically alert users of system malfunction or failure or loss of acoustic coupling without the assistance of trained US practitioners.
5. AI/ML methods are likely to play an important role in the reconstruction, analysis, and interpretation of wearable US data. Additional research is needed in understanding the behavior of these methods in unexpected scenarios not encountered when training the AI/ML models.

## VI. Conclusion

Wearable US has the potential to revolutionize medical imaging by significantly improving the accessibility and diagnostic and monitoring capability of US imaging. Wearable US can simplify clinical US imaging workflow and improve diagnostic performance of US in both traditional and new imaging settings, as well as nondiagnostic biosensing applications such as biofeedback and human–machine interfaces. Neuromodulation and therapy are other important applications not discussed in this article. As the field of wearable US is rapidly growing, researchers, developers, and users should be aware of important safety considerations to prevent adverse bioeffects associated with tissue heating and skin irritation. They should be well informed on the current safety and QC standards of US devices. The field of wearable US is ripe for new advances in instrumentation, device design, and advanced data analytic capabilities based on AI/ML. Clinical translation and adoption of the technology need to be facilitated by a deep and early engagement with prospective users of wearable US technology who will provide key insights for researchers and developers to come up with practical and viable solutions for unmet clinical needs.

## Figures and Tables

**TABLE I:** Summary of Example Applications of Wearable Ultrasound

Applications	Desired measurements and relevant wearable US imaging modes	Advantages of wearable ultrasound	Application-specific challenges	Common barriers for wearable ultrasound devices
Cardiovascular	Measurements: Blood pressure waveform, blood flow velocity, heart rate, vessel morphology, pulse wave velocity, heart wall dynamics, chamber volume/dimension, cardiac output, resistive index, etc. Imaging modes: A-mode, B-mode, M-mode, color flow, color power, PW Doppler	Cardiovascular assessment during exercise. Long-term and continuous monitoring of cardiovascular status. Reduce variability of echocardiographic metrics due to patient positioning.	Demanding imaging conditions that require large imaging depth of penetration and high frame rate. Challenging to achieve good cardiac imaging quality in patients with large body habitus. Large cardiac movement is challenging to separate from weak blood flow signal (i.e., clutter rejection). Large body movement during applications such stress echo.	Lower imaging resolution and contrast as compared to standard ultrasound imaging devices. Challenges associated with continuous acoustic coupling between the probe and the skin, especially during free movement. Concerns about skin health for long-term use. Concerns about thermal effects for long-term continuous use. Challenges associated with varying sensor positions and resulting acoustic field of flexible arrays during body movement. Noise, device measurement accuracy, and device durability considerations under body movement. Data analysis and interpretation, distinguishing actionable data from noise. Integration into clinical workflow such as training, clinician and patient education, costs, and quality control. Large-scale clinical trials are necessary for establishing advantage of wearable US over existing standard of care.
Transcranial imaging	Measurements: Blood flow velocity and waveform Emboli monitoring Brain motion and acceleration Imaging modes: Transcranial PW Doppler	Long-term and continuous monitoring of cerebrovascular status. Less rigid device that adheres to the skin, reducing discomfort to patients. Reduced operator dependency with electronic steering and automatic vessel search and tracking.	Strong attenuation and phase aberration induced by the skull. Head movement, and brain movement relative to the skull. High acoustic power needed and associated power consumption.	
Fetal monitoring	Measurements: Fetal movement, heart rate. Imaging modes: A-mode, B-mode, M-mode, PW and CW Doppler	Continuous monitoring of fetal heart rate before and during labor for high-risk pregnancies. Can be performed by non-expert users in outpatient settings. May be more accessible and affordable in underserved communities to prevent stillbirths.	Acoustic dose and potential for bioeffects.	
Musculoskeletal, rehabilitation and neuroengineering	Measurements: Muscle force, volitional intent, bilateral asymmetry, muscle synergy, muscle tissue properties, muscle architecture Imaging modes: A-mode, B-mode, M-mode, PW Doppler	Easy to maintain probe position. Continuous and long-term monitoring of muscle function over time and during exercise and other activity. Real-time imaging feedback during body movement with the opportunity to develop new quantitative biomarkers for exercise dosing, rehabilitation assessment, and characterizing muscle activation patterns for specific and repeatable movement tasks. Provides spatially resolved muscle and motor unit measurements that are more specific and robust than EMG.	Body movement that can be rapid, frequent, and with large range. Quantitative measurements need to be validated, since US is not commonly used in current practice.	

**TABLE II:** Maximum Exposure Durations Recommended by AIUM

TI	Obstetric, Neonatal Transcranial, Neonatal Spinal	Other except eye
> 6.0	0	0
5.0 – 6.0	0	< 15 s
4.0 – 5.0	0	< 1 min
3.0 – 4.0	0	< 4 min
2.5 – 3.0	< 1 min	< 15 min
2.0 – 2.5	< 4 min	< 1 hour
1.5 – 2.0	< 15 min	< 2 hours
1.0 – 1.5	< 30 min	No limit
0.7 – 1.0	< 60 min	No limit
< 0.7	No limit	No limit

The first category (Obstetric …) includes gynecologic when pregnancy is possible. The Other category includes adult transcranial, general abdominal, peripheral vascular, neonatal (except head and spine) and other scanning examinations except the eye. For obstetric exams, monitoring the TIS is recommended up to 10 weeks from the last menstrual period (LMP) or a crown rump length (CRL) of about 33–34mm, and TIB thereafter. Dwell times should be reduced by 33% for ARFI and pulsed Doppler examinations when bone is near the transducer focus[90], [95]. Table reprinted from[90].

## Highlights

- Wearable ultrasound is poised to push the use of ultrasound technology beyond traditional diagnostic settings and will provide opportunities to engage clinicians, scientists, and engineers from a broad range of disciplines.
- Wearable ultrasound applications will require development and validation of robust quantitative biomarkers that provide actionable information about tissue properties, physiology, and function.
- Future development of wearable ultrasound systems should be guided by engineering and technological considerations as well as user needs and considerations of factors that impact safety and data quality.
